# A Particular River-Whiting Phenomenon Caused by Discharge of Hypolimnetic Water from a Stratified Reservoir

**DOI:** 10.1371/journal.pone.0137860

**Published:** 2015-09-11

**Authors:** Jingan Chen, Haiquan Yang, David Dian Zhang, Dan Xu, Jing Luo, Jingfu Wang

**Affiliations:** 1 State Key Laboratory of Environmental Geochemistry, Institute of Geochemistry, Chinese Academy of Sciences, Guiyang 550002, China; 2 Department of Geography, The University of Hong Kong, Pokfulam Road, Hong Kong, China; 3 College of Resources and Environmental Engineering, Guizhou University, Guiyang 550025, China; Centro de Investigación Científica y Educación Superior de Ensenada, MEXICO

## Abstract

A particular river-whiting phenomenon occurred in the early 2000s in the Xiaoche River and since then it has been reoccurring from June to November each year. Residents were surprised by this phenomenon and worried about it. This study was designed to reveal the forming mechanism of the river-whiting phenomenon. A comparison of T, EC, ORP, DO, TDS and δ^34^S in the culvert water and discharge pipe water with that in the water column of Aha Reservoir strongly indicated that the culvert water and discharge pipe water derived primarily from the hypolimnetic reservoir water. When the hypolimnetic water enriched in SO_4_^2-^ and H_2_S, through seepage from the penstock, flows into the Xiaoche River, the water's supersaturation degree with respect to CaSO_4_ is increased as a result of increased temperature and DO, thus colloid CaSO_4_ can be formed. This is the essential cause of the river-whiting phenomenon. The sources of high concentrations of SO_4_^2-^ and H_2_S in hypolimnetic water include not only direct SO_4_^2-^ and H_2_S input of acid mine drainage as a result of irrational coal mining in the watershed, but also the sulfur-enriched surface sediments which may release H_2_S through the sulfate reduction processes. The contaminated sediment has acted as an important contamination source for sulfur to the overlying water in Aha Reservoir. There are more than 50,000 large dams in the world until now. With the increase of reservoir age and the persistent accumulation of pollutants within the reservoir system, discharged hypolimnetic water may contain high levels of pollutants and lead to unpredicted disasters. More investigations are needed to illuminate the water quality condition of discharge water from reservoirs and estimate its impacts on the downstream eco-environment.

## Introduction

The damming of rivers has been one of the most dramatic and widespread, deliberate impacts of man on the natural environment [1]. There are about 50000 large dams (>15m in height) and more than 800000 small dams in the world until now [2, 3]. Problems associated with river impoundment include increased incidence of earthquakes and landslides, fragmented natural habitat, decreased river flow-velocity, deteriorated water quality, reduced aquatic and terrestrial biodiversity, as well as eco-environmental changes downstream from dams [1, 4–7]. Although these problems have been noted and widely investigated recently, we may not, even now, understand fully the range and magnitude of the induced changes associated with river impoundment. Actually, some changes can be only detected after several decades of the damming. With the increase of reservoir age and the persistent accumulation of pollutants within reservoir system, discharge water from the hypolimnion may contain high levels of pollutants released from sediments in strong reducing conditions. This may lead to unpredicted disasters.

Over the past decades, most environmental investigations about reservoirs have focused on the unique biogeochemical cycle and consequent eco-environmental effects within the reservoir system, while little attention has been paid to the water quality of discharge water from reservoirs and its impacts on the downstream eco-environment. In this study, we, for the first time, reported a particular river-whiting phenomenon in the Xiaoche River caused by discharge of hypolimnetic water from a seasonally stratified reservoir, which occurred four decades after the reservoir impoundment. Although the whiting phenomenon has been noted in the Xiaoche River for several years, there is no definite explanation for its occurrence until now. In fact, whiting phenomena is very common in estuaries and oceans, which is normally attributed to bio-induced calcite precipitation or re-suspended sediment. The aim of this study was to reveal the essential reason of the whiting phenomenon in the Xiaoche River, and to discover the differences or similarities in comparison to the whiting phenomenon in estuaries and oceans.

## Study Area and River-Whiting Phenomenon

### Study area

**Fig 1:**
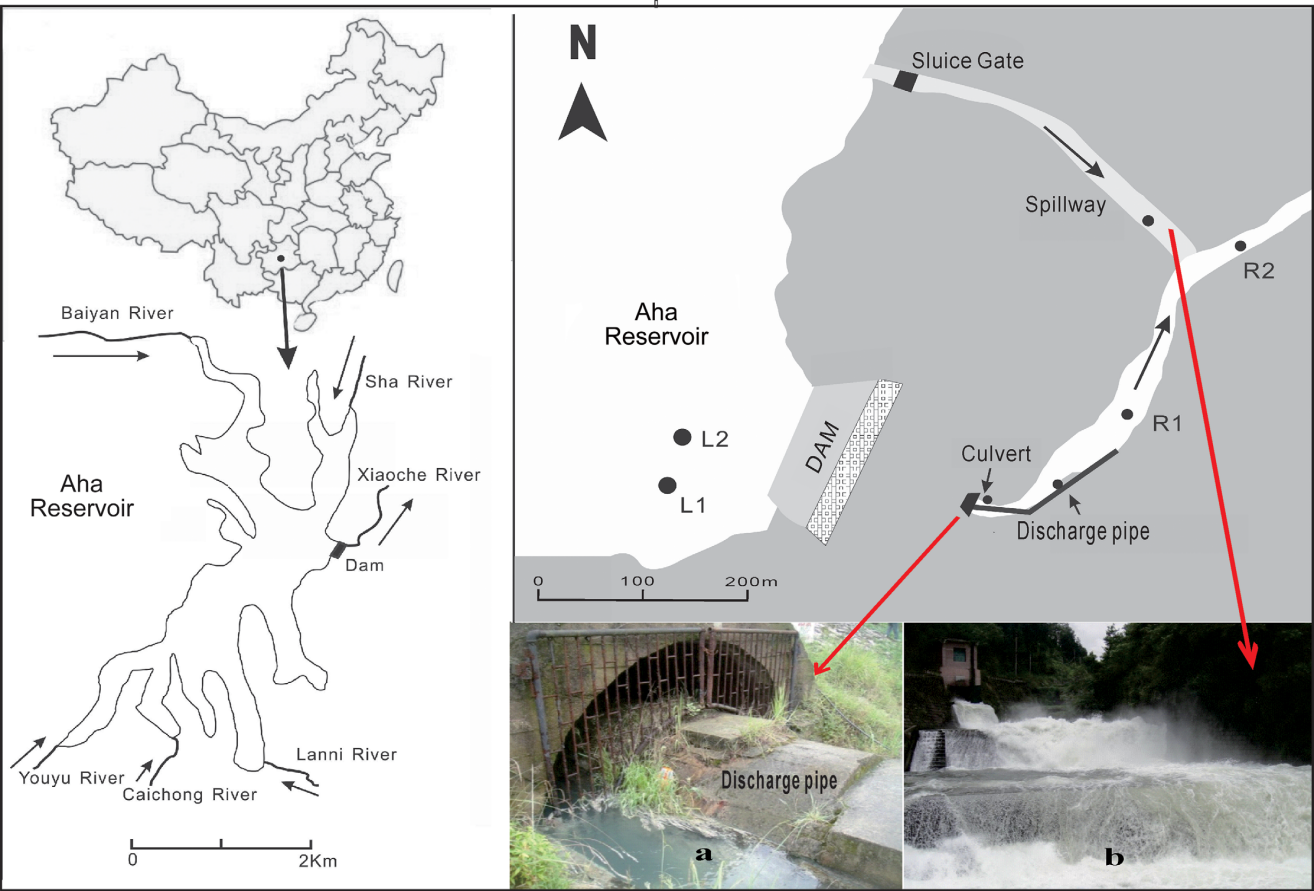
Illustration of Aha Reservoir and the Xiaoche River showing sampling locations. (a) photo of the culvert with discharge pipe inside. (b) photo of the spillway. L1 and L2 represent two sampling sites in Aha Reservoir; R1 and R2 represent two sampling sites in the Xiaoche River.

Aha Reservoir (106°37′∼106°40′ E, 26°30′∼26°34′ N) is located in the suburb of Guiyang, the capital of Guizhou Province, southwestern China (Fig 1). It was constructed in the upstream of the Xiaoche River for the purpose of irrigation, drinking-water supply, flood control and tourism in 1960. It has a watershed area of 190 km^2^, a surface area of 4.5 km^2^, a volume of 5.42×10^7^ m^3^, an average depth of 13 m and maximum depth of 26 m [8]. The regional climate in Guiyang is mainly influenced by the southeast monsoon, with lesser influence by the southwest monsoon. Thus it is obviously seasonal. More than 80% of the mean annual precipitation of 1109 mm falls between May and October, when warm-humid air from the southeast and southwest predominates. From November to April, cold-dry air from the north prevails and there is less precipitation. The average annual evaporation is 932 mm. The mean annual temperature is 15.3°C. The highest monthly average temperature of 23.6°C occurs in July while the lowest of 4.9°C occurs in January. Five tributaries including the Youyu River, Caichong River, Lannigou River, Baiyan River and Sha River, flow into Aha Reservoir (Fig 1) with an average annual flow of 1.04×10^8^ m^3^ within 2000–2010. The water residence time in Aha Reservoir is about 0.5 a.

The bedrock in the catchment consists mainly of Permian carbonate rock and coal-bearing strata, covered with silico-alumina and silico-ferric yellow soil [8–9]. The terrain around Aha Reservoir is heavily vegetated. There are more than 200 small coal mines widely distributed in the watershed. A large amount of acid mining drainage was discharged to the local environment due to oxidation of sulfides-containing coal in the process of mining, and flowed finally into Aha Reservoir during the past three decades, especially in the 1980s-1990s. Persistent input of acid mining drainage has resulted in a series of environmental accidents in Aha Reservoir since the end of the 1990s. The reservoir water turned black and fish kills occurred after sudden mixing [8, 10]. A large number of studies have been carried out to investigate the biogeochemical cycling of heavy metals and sulfur within the reservoir [8–15], but less attention was paid to the released water and its effects. There are two water outlets in the reservoir (Fig 1). One is the spillway which releases the surface water of Aha Reservoir. The other one is the culvert penstock which is located just off the bottom in front of the dam and releases hypolimnetic water when necessary. These two outlets became the main water sources of the Xiaoche River after Aha Reservoir was initially impounded in 1960. The Xiaoche River has an average width of 8 m. It, as a tributary of the Nanming River, drains ultimately into the Yangtze River.

### River-whiting phenomenon

**Fig 2:**
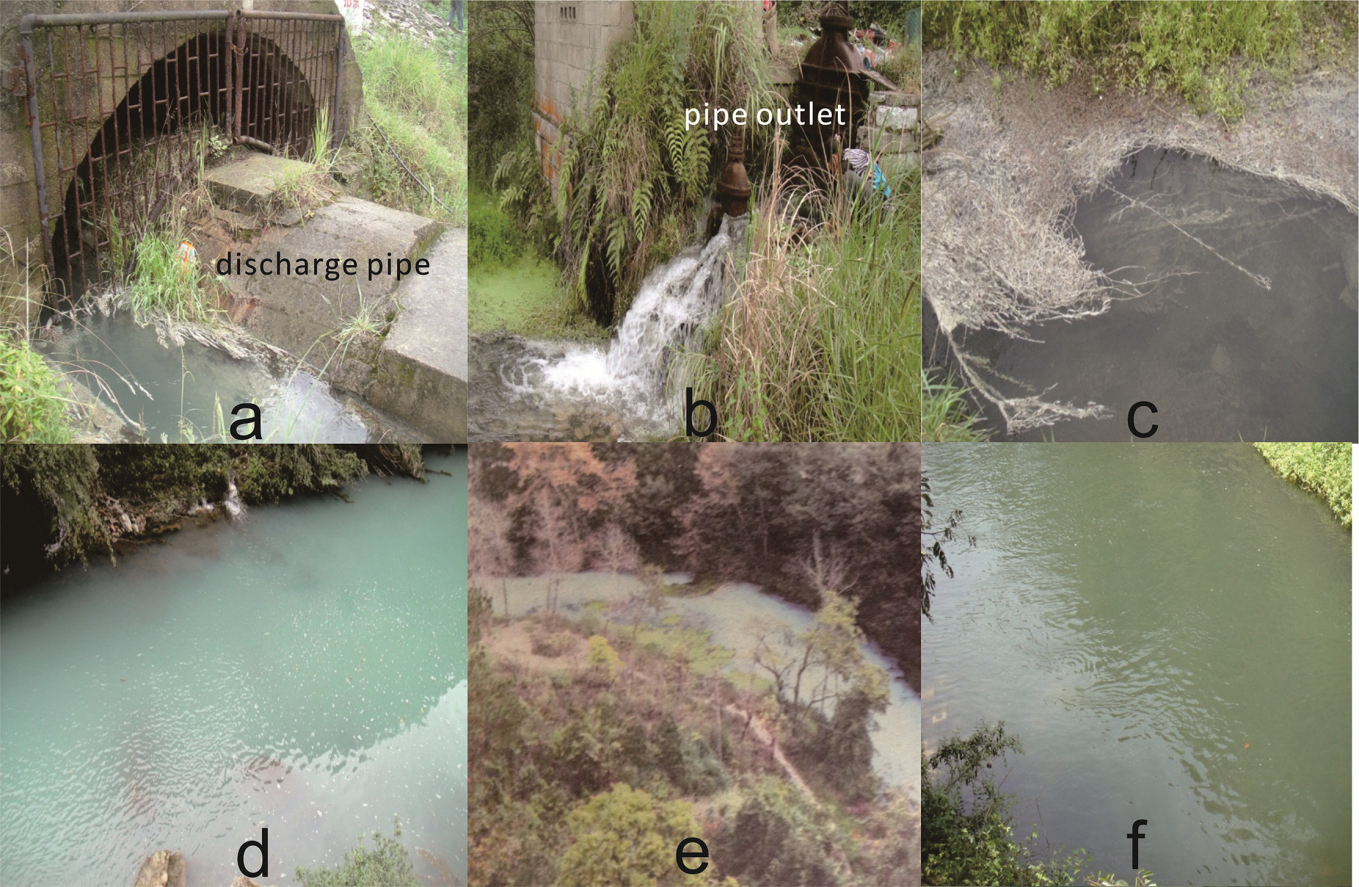
Photos of the Xiaoche River. (a) the culvert water. (b) the discharge pipe water. (c) plants attached with white material. (d) milky-white and turbid river water. (e) photo showing river-whiting phenomenon in Qianzhong Morning Post [17]. (f) river-whiting phenomenon disappearing gradually downstream.

A strange river-whiting phenomenon occurred in the headstream of the Xiaoche River in the early 2000s, and since then it has been reoccurring from June to November each year. The water flowing out from the culvert sent out a smell of rotten eggs and turned gradually milky-white and turbid in the Xiaoche River, like dirty soap water. This phenomenon developed to the highest extent about 500 meters away from the dam and persisted 2km downstream (Fig 2). Plants in the river were attached with white material. Residents were surprised by the river-whiting phenomenon and worried about it. Underground water input was supposed to be the cause of river-whiting in a previous investigation [16]. However, this supposition could not explain either why the river-whiting phenomenon occurred only in recent ten years or why it appeared seasonally. Other potential factor possibly contributed to river-whiting includes reservoir water seeping through the culvert penstock which is located just off the bottom. Subsequently, this study was designed to reveal the forming mechanism of the river-whiting phenomenon in the Xiaoche River, and to determine if water released from Aha Reservoir could be a primary cause of river-whiting.

## Sample Collection and Analysis

### Sampling

The Management Office of Aha Reservoir invited us to conduct this study and issued the permission for the sampling activity in the research area. There are three main water sources in the headwater area of the Xiaoche River. The first source is water flowing out from the culvert at the bottom of the dam. Inside the culvert, lies a discharge pipe (Fig 2a) which is connected to the hypolimnion of Aha Reservoir through a penstock located just off the bottom. The culvert water includes the hypolimnetic reservoir water seeping through the penstock and possible underground water. It has a flux of about 12, 000 m^3^/a. The second source is water from the discharge pipe inside the culvert, flowing into the Xiaoche River at the pipe outlet (Fig 2b) which is located about 100 m downstream from the culvert. The only source of the discharge pipe water is the hypolimnetic water of Aha Reservoir seeping through the penstock. It has a flux of about 2, 000 m^3^/a. The third source is the surface water of Aha Reservoir released from the spillway intermittently (Fig 1c), draining into the Xiaoche River at a confluence site about 600 m away from the dam.

The culvert water, discharge pipe water and spillway water were sampled in September 2012. At the same time, river water sample R_1_ was collected where the river-whiting was the most prominent (Fig 2d and 2e), and R_2_ was collected at a downstream site where the river-whiting phenomenon disappeared gradually (Fig 2f). White material covered on the plants in the river was also collected for analyzing its chemical and mineral compositions.

Sampling stations L1 and L2 close to the dam (Fig 1) were chosen in Aha Reservoir to collect water samples at different depths (from 0.5 m to 26 m) using an acid-cleaned, Teflon lined, 10-L Nisiki sampler. Two sampling campaigns were conducted in September 2012 and March 2013 to represent the stratified and mixing regimes, respectively, and to investigate the variations of physico-chemical properties in the water column. All sampling vessels including polyethylene bottles were cleaned with acid in laboratory and pre-rinsed with the corresponding water samples for three times in field. Sediment cores were collected at sampling station L2 using a SWB-1 gravity sampler [18] in September 2012. The water-sediment interface was not disturbed during coring and the sediment cores were perfectly preserved. Sediments were immediately divided into 5–10 cm sections and put into plastic bags in the field. Sediment samples were immediately transferred to the laboratory in iceboxes (<4°C) and freeze-dried. Afterwards, the samples were ground and sieved with a standard 100-mesh sieve for chemical analysis.

### Analytical techniques

A multi-parameter water quality sonde (YSI 6600 V2) was used for determining the pH, water temperature (T), electrical conductivity (EC), oxidation-reduction potential (ORP), dissolved oxygen (DO) and total dissolved solids (TDS) immediately after sampling.

Water samples for anion and cation analysis were filtered with 0.45 μm membrane filters in field and the filtered samples for the measurement of cations were acidified to pH<2 with distilled HNO_3_ immediately. Concentrations of Cl^-^ and SO_4_^2-^ were determined by ion chromatography (Dionex ICS-90) within 24 hours of sampling. inductively coupled plasma optical emission spectroscopy (ICP-OES, Vista MPX) was used to determine the concentrations of K^+^, Na^+^, Ca^2+^, Mg^2+^.

Water samples for the measurement of sulfur isotopic compositions were filtered through a 0.45 μm Millipore HA membrane filter within 24 hours after sampling and were acidified to pH<2. Dissolved sulfate was recovered as BaSO_4_ after the addition of 10% BaCl_2_. The precipitation of BaSO_4_ was rinsed with Milli-Q water (18.2 MΩ) until there was not Cl detected and the precipitation was then combusted at 800°C in a muffle furnace for 2 hours. The sulfur isotopic compositions were determined by IsoPrime CF-IRMS with NBS-127 and GBW04415 as reference standards at the State Key Laboratory of Environmental Geochemistry, Chinese Academy of Sciences. The results were reported as δ^34^S in part per thousand deviations relative to the Vienna Cañon Diablo Troilite (V-CDT) standard with a reproducibility of ±0.2‰.

Total sulfur contents in sediment samples were determined by elemental analyzer (Vario Macro Cube). The mineral compositions of the white material were identified by X-ray Diffractometer (D/Max-2200) at the State Key Laboratory of Ore Deposit Geochemistry, Chinese Academy of Sciences.

## Results and Discussion

### Material sources of river-whiting

The spillway water is believed to have nothing to do with the forming of the river-whiting phenomenon (RWP) because of the following reasons. Firstly, RWP has occurred in the upstream section before the spillway water flows into the Xiaoche River. Secondly, RWP disappears immediately downstream from the convergence site when the spillway gate is opened and the surface reservoir water mingles into the riverine water, while RWP persists 2km downstream when the spillway gate is closed and no surface reservoir water is released. Consequently, RWP in the Xiaoche River may be caused by the culvert water and/or the discharge pipe water. As described before, the discharge pipe water consists entirely of the hypolimnetic water of Aha Reservoir while the culvert water includes the hypolimnetic reservoir water and possible underground water. The culvert water and discharge pipe water were analyzed and compared simultaneously to judge whether there was underground water input in the culvert water. The result showed that the culvert water had almost the same physico-chemical characteristics as the discharge pipe water (Table 1), suggesting that the culvert water comes primarily from the hypolimnetic reservoir water, with negligible underground water input. This was verified by the sulfur isotopic compositions of the SO_4_^2-^ in the culvert water, with a δ^34^S value of 7.00‰ similar to that of 7.14‰ in the discharge pipe water. It was shown in Table 1 that the culvert water has a little more positive ORP and slightly higher DO than the discharge pipe water. This may result from the earlier exposure of the culvert water to air outside than the discharge pipe water.

**Table 1:** The physico-chemical characteristics of the culvert water, discharge pipe water, spillway water and river water.

Samples	T (°C)	EC(ms/s)	ORP (mV)	DO (mg/L)	pH	TDS(g/L)	Ca^2+^ (mg/L)	K^+^ (mg/L)	Mg^2+^ (mg/L)	Na^+^ (mg/L)	Cl^-^ (mg/L)	SO_4_^2-^ (mg/L)
Culvert water (Fig 2a)	9.5	0.564	-359.6	1.51	7.60	0.523	103.18	4.24	20.30	8.55	11.97	200.30
Discharge pipe water (Fig 2b)	9.3	0.566	-399.8	0.17	7.53	0.529	100.02	4.15	20.16	8.56	12.30	204.56
River water R_1_ (Fig 2d)	15.1	0.589	-195.2	3.66	7.80	0.536	78.78	4.38	19.14	8.42	9.97	190.88
Spillway water (Fig 1b)	22.8	0.610	-51.2	9.20	7.94	0.442	76.63	4.26	17.27	7.41	9.73	166.41
River water R_2_ (Fig 2f)	22.7	0.581	-42.9	6.01	7.88	0.427	64.16	4.21	17.38	8.24	9.51	158.60

**Fig 3:**
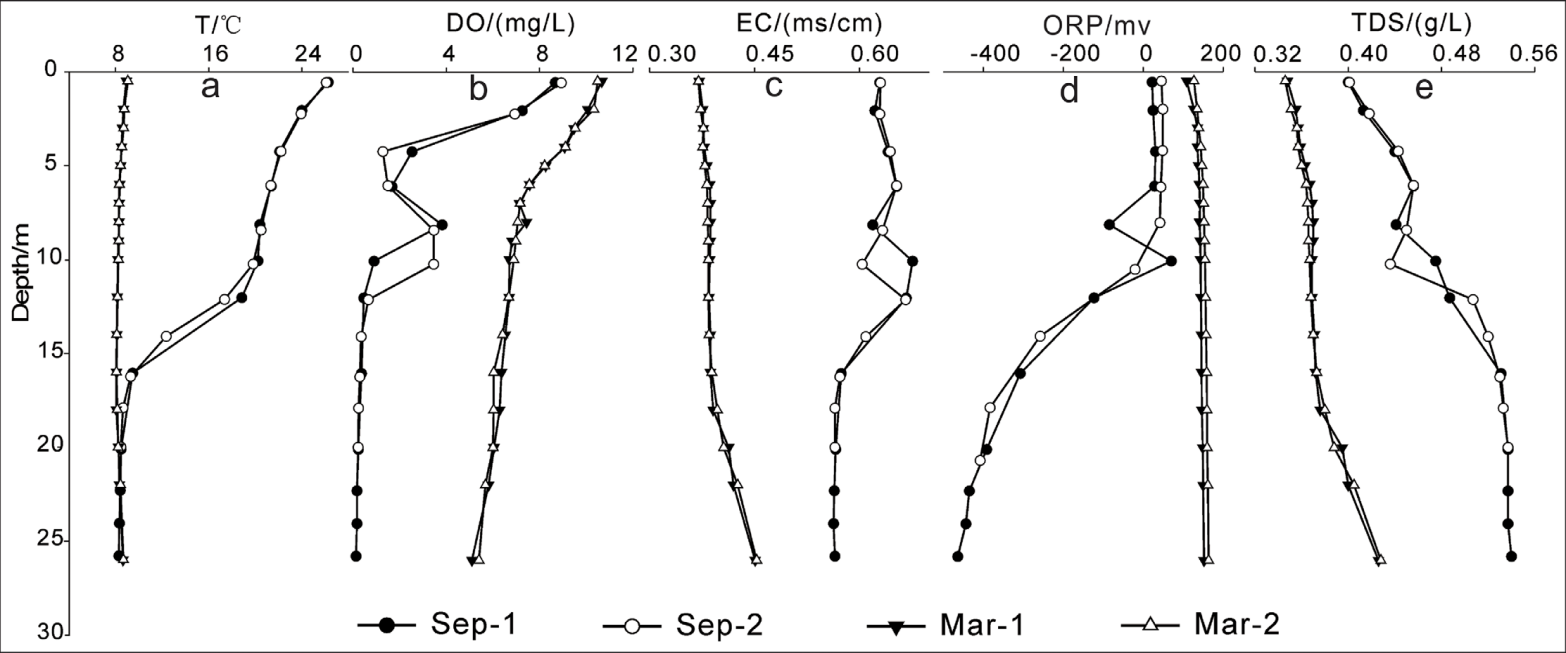
Variations of T、EC、ORP、DO、TDS in the water column of Aha Reservoir in September (stratified period) and March (non-stratified period). Sep-1 and Mar-1 represent reservoir water at station L1 sampled in September and March, respectively; Sep-2 and Mar-2 represent reservoir water at station L2 sampled in September and March, respectively.

In order to further testify that the hypolimnetic water of Aha Reservoir was the main source of the culvert water and discharge pipe water, vertical profile variations of physico-chemical properties in the water column were compared with that in the culvert water and discharge pipe water (Table 2 and Fig 3). The reservoir was clearly stratified in summer for T, EC, ORP, DO and TDS with an anoxic cool hypolimnion about 10 m thick extending from 16 m depth to the bottom at 26 m (Fig 3). There is strong evidence suggesting that the hypolimnetic reservoir water in front of the dam was the primary source of the culvert water and discharge pipe water. The temperature of the culvert water and discharge pipe water ranged between 9.3°C and 9.5°C, which was accordant with the temperature of the hypolimnetic reservoir water with a depth larger than 15m (Fig 3a). The conductivity in the culvert water and discharge pipe water varied from 0.564 to 0.569 ms/cm. From Fig 3c, it can be inferred that the discharged water should come from the hypolimnion with a depth of between 18 m and 26 m. Similarly, the vertical variations of ORP and DO in the water column (Fig 3b and 3d) suggested the depth of the discharged water was larger than 18 m. TDS profile (Fig 3e) indicated the discharged hypolimnetic water lies between 15 m and 26 m. Taken together, the variations of T、EC、ORP、DO and TDS in the water column of Aha Reservoir strongly suggested that the culvert water and discharge pipe water came from the hypolimnetic reservoir water between 18 m and 26 m, which seeped through the penstock located just off the bottom.

**Table 2:** Hydrochemical compositions in the water column, culvert water, spillway water and discharge pipe water.

Sample	Ca^2+^ (mg/L)	K^+^ (mg/L)	Mg^2+^ (mg/L)	Na^+^ (mg/L)	Cl^-^ (mg/L)	SO_4_^2-^ (mg/L)
Culvert water	103.18	4.24	20.30	8.55	11.97	200.30
Discharge pipe water	100.02	4.15	20.16	8.56	12.30	204.56
Spillway water	76.63	4.26	17.27	7.41	9.73	166.41
RWS-0.5m	64.56	4.24	16.24	7.48	10.68	168.10
RWS-2m	63.90	4.11	15.55	7.22	10.35	165.51
RWS-4m	74.25	4.69	16.20	7.98	11.15	162.00
RWS-8m	89.90	3.12	14.50	7.50	7.46	165.44
RWS-12m	98.28	3.27	15.94	6.74	8.06	194.08
RWS-16m	97.55	3.15	15.15	5.86	7.81	181.71
RWS-20m	96.51	3.99	19.83	8.07	11.08	201.39
RWS-26m	98.11	4.28	20.73	8.49	11.68	200.40
RWM-0.5m	98.52	5.19	17.78	9.01	8.78	173.78
RWM-15m	98.11	5.11	17.32	8.89	9.11	166.28
RWM-25m	98.72	5.30	17.75	9.26	9.20	166.04

RWS represents reservoir water sampled in September; RWM represents reservoir water sampled in March.

The culvert water and discharge pipe water were characterized by high levels of Ca^2+^ and SO_4_^2-^ and low concentrations of Mg^2+^, K^2+^, Na^+^ and Cl^-^ (Table 2). In the water column, concentrations of Ca^2+^ and SO_4_^2-^ increased with the depth while concentrations of other ions kept stable with only small fluctuations. It is easy to be seen from Table 2 that concentrations of Ca^2+^, SO_4_^2-^, Mg^2+^, K^2+^, Na^+^ and Cl^-^ in the hypolimnion between 20–26 m were quite close to that of the culvert water and discharge pipe water, which also proved the primary contribution of the hypolimnetic water to the culvert water and discharge pipe water.

From the above discussions, it can be concluded that the hypolimnetic water of Aha Reservoir provide the ultimate materials for causing the river-whiting phenomenon in the Xiaoche River.

### Forming mechanisms of river-whiting phenomenon

The earlier investigation by Guizhou Normal University has sampled the reservoir water at depths of 0, 8, and 15 m. Temperature and DO concentrations of the reservoir water were measured and compared with that of the culvert water [16]. A considerable difference in temperature between the reservoir water and the culvert water was found, according to which the reservoir water was supposed to have negligible contribution to the culvert water and have nothing to do with RWP [16]. The investigation proposed that RWP in the Xiaoche River was possibly due to input of sulfur-enriched underground water in the culvert. On the one hand, the investigation has sampled only the upper water between 0–15 m, overlooking the deeper hypolimnetic water, thus it was incomplete and the inference was unreliable. On the other hand, the investigation could not explain either why RWP occurred seasonally or why RWP appeared only in recent ten years.

In this study, all kinds of evidences indicated that the hypolimnetic water of Aha Reservoir was the primary source of the culvert water and discharge pipe water, and resulted in RWP in the Xiaoche River. In order to further understand the forming processes and mechanisms of RWP, the white materials in the river were analyzed.

**Fig 4:**
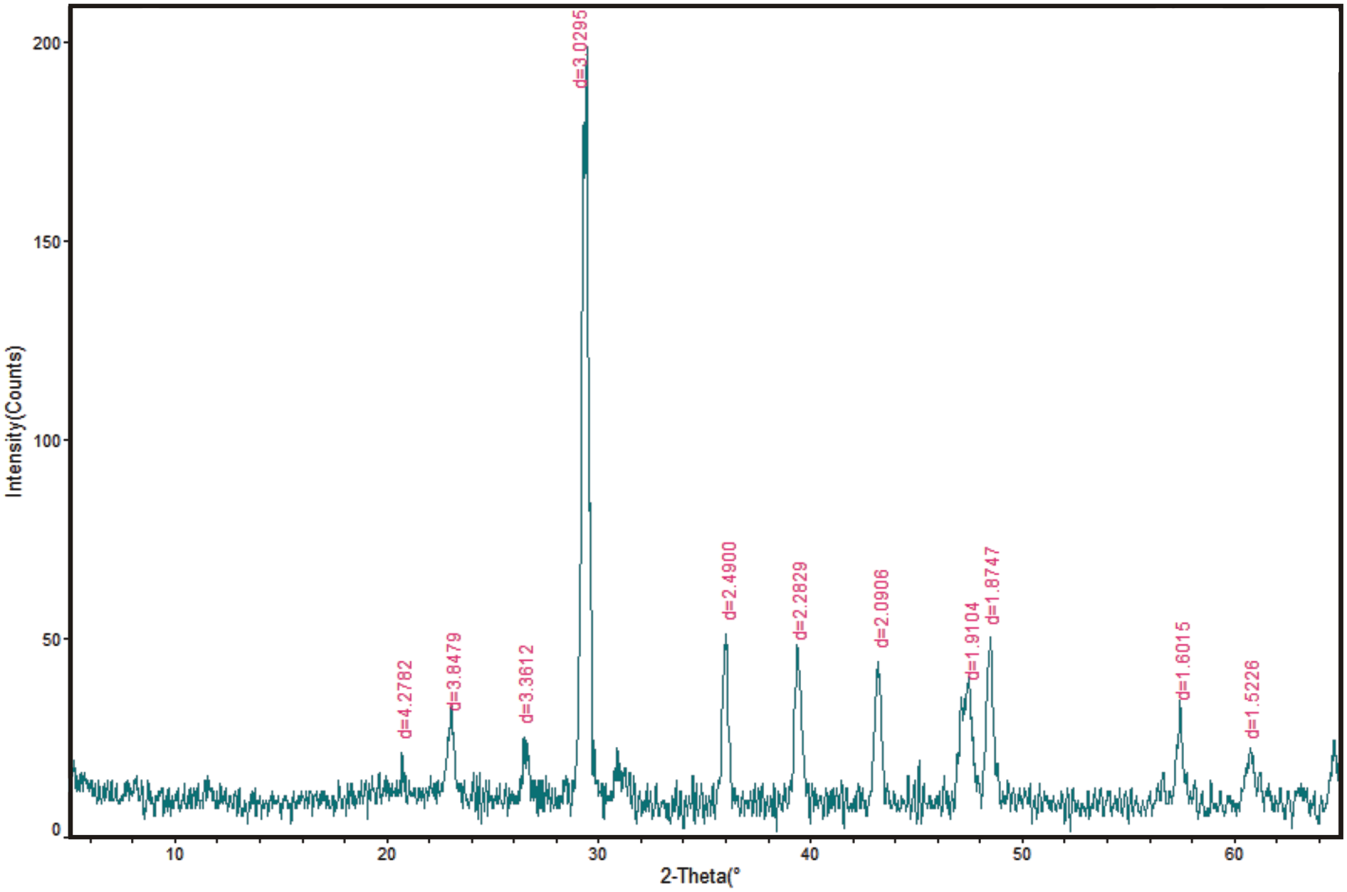
X-ray diffraction spectrum of the white materials in the Xiaoche River.

X-ray diffraction spectrum (Fig 4) shows that the white materials consist primarily of amorphous gypsum (CaSO_4_) and calcite (CaCO_3_). The forming processes of CaSO_4_ can be represented simply by the following reaction:

$$Ca^{2+}+SO_{4}^{2-}=CaSO_{4}↓$$

The precipitation of CaSO_4_ requires supersaturation with respect to CaSO_4_ in solution. A conventional method of judging the water's supersaturation with respect to CaSO_4_ is to compare the ionic activity product (IAP) with the equilibrium constant (K_e_). The culvert water and the discharge pipe water were characterized by high concentration Ca^2+^ and SO_4_^2-^ (Table 2). According to the hydrochemical data of the Xiaoche River (Tables 1 and 2), the supersaturation indexes of the water (IAP/K_e_) with respect to CaSO_4_ were calculated to vary between 0.58 and 16.97, implying CaSO_4_ precipitation can be produced in the river. CaSO_4_ in water exists as a white colloid substances, so the river turns milky-white and turbid. This is the essential cause of the river-whiting phenomenon in the Xiaoche River. It is quite different from the whiting phenomenon in estuaries and oceans which is normally caused by bio-induced calcite precipitation or re-suspended sediment.

### Why did the river-whiting phenomenon occur seasonally?

Previous investigation showed that seasonal thermal stratification existed in Aha Reservoir normally from June to October [10, 13]. As shown in Fig 3, there is thermal stratification in September and no stratification in March. An anoxic hypolimnion is developed as a result of a stratification regime and causes notable changes in the water quality of the hypolimnion. It is well known that water below the thermocline is normally depleted of dissolved oxygen, and contains high concentrations of SO_4_^2-^ and hydrogen sulfide produced during the process of anaerobic decomposition, and elevated levels of metals such as iron and manganese brought into solution from the bottom sediments as a result of the strong reducing conditions [1, 8, 10, 19]. The water discharged from the reservoir hypolimnion to the culvert and discharge pipe is markedly anoxic and enriched in sulfur during June-October (Tables 1 and 2). When the culvert water and discharge pipe water flow into the Xiaoche River, the water's supersaturation degree with respect to CaSO_4_ will be increased quickly and colloid CaSO_4_ can be formed because of the following reasons. Firstly, DO in the water will rise as a result of the water exposure to air outside, which leads to the oxidation of hydrogen sulfide and the formation of SO_4_^2-^. This will increase the ionic activity product of Ca^2+^ and SO_4_^2-^ in the river. Secondly, the solubility and dissolution equilibrium constant of CaSO_4_ are lower at high temperature than at low temperature. When the culvert water and discharge pipe water flow into the Xiaoche River, the water temperature goes up gradually from below 9.5 to above 22, thus, the water’s supersaturation degree with CaSO_4_ is increased gradually with increasing water temperature, promoting the precipitation of CaSO_4_ in the river. This is why the river-whiting phenomenon occurred in the Xiaoche River from June to October. From November to next May, Aha Reservoir is usually mixed well (Fig 3), and the bottom water has lower levels of SO_4_^2-^ compared to June-October (Table 2). Furthermore, the lower water temperature in the Xiaoche River in winter is conducive to CaSO_4_ dissolution. Consequently, the river-whiting phenomenon hardly occurs during November-May.

### Why did the river-whiting phenomenon appear only in recent ten years?

**Fig 5:**
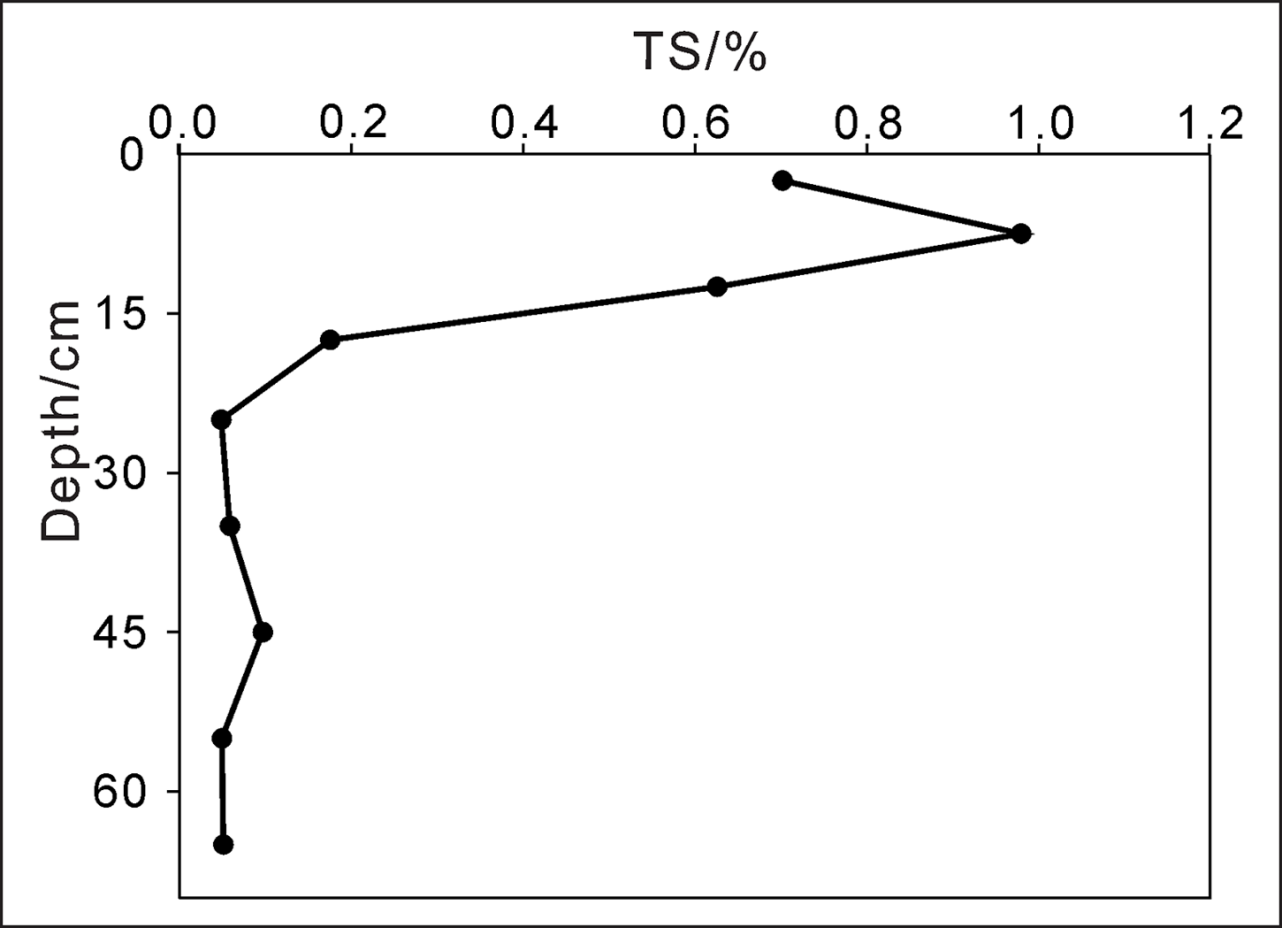
Profile variations of the sulfur contents in the sediment of Aha Reservoir.

As discussed before, the hypolimnetic reservoir water enriched in sulfur provide primary material source for the formation of the river-whiting phenomenon in the Xiaoche River. The sources of high concentrations of SO_4_^2-^ and H_2_S in the hypolimnetic water include not only the direct SO_4_^2-^ and H_2_S input from the catchment, but also the rich sulfur in surface sediments which may release H_2_S during the process of anaerobic decomposition as a result of the strong reducing conditions in stratified period [8, 10, 13]. In the sediment profile of Aha Reservoir, the sulfur contents kept stable with only small fluctuations between 0.05% and 0.1% in sediments below 20 cm, and then it increased rapidly from 0.1% at depth of 20 cm to 1% at depth of 10 cm with the decrease in sediment depth, followed by a decline from 10 cm to the sediment-water interface (Fig 5). The profile variations of the sulfur contents in Aha sediments recorded truly the pollution history in the catchment. It is well known that acid mine drainage (AMD) with high concentrations of sulfur and heavy metals was produced by the coal mining activities in the watershed, and has been discharged directly or indirectly to Aha Reservoir since the beginning of the 1980s, reaching the discharge peak in the middle of the 1990s [8, 10, 13, 15]. This was well reflected by the rapid sulfur increase from 20 cm to 10 cm in the sediment profile. According to the sedimentation rate in Aha Reservoir [20, 21], sediments at depths of 20 cm and 10 cm were corresponding to 1980 and 2000, respectively. The coal mines in the catchment were closed gradually after 1990s and the input of acid mine drainage became less and less. This, together with the sulfate reduction processes caused by sulfate reducing bacterial in the surface sediments, led to the decrease in sulfur contents in the uppermost 10 cm sediments. The contaminated sediment, as a result of persistent acid mine drainage input from the watershed, has acted as an important contamination source for sulfur to the overlying water, especially from the beginning of 2000s. This is why the river-whiting phenomenon occurred only in recent ten years.

Concentrations of SO_4_^2-^ in Aha Reservoir are about two times of that in Hongfeng Lake which is only 30 km away from Aha Reservoir. There are much less coal mining sites in the watershed of Hongfeng Lake. Thus, the serious sulfur pollution in Aha Reservoir should be caused by local coal mining activities within the reservoir watershed instead of regional pollution. Persistent discharge of acid mine drainage as a result of irrational coal mining is the ultimate cause of sulfur-enriched reservoir water and the river-whiting phenomenon in the Xiaoche River.

## Conclusions

A comparison of T, EC, ORP, DO, TDS and δ^34^S in the culvert water and discharge pipe water with that in the water column of Aha Reservoir strongly indicated that the culvert water and discharge pipe water derived primarily from the hypolimnetic reservoir water. When the hypolimnetic water enriched in SO_4_^2-^ and H_2_S, through seepage from the penstock, flows into the Xiaoche River, the water's supersaturation degree with respect to CaSO_4_ is increased as a result of increased temperature and DO, thus colloid CaSO_4_ can be formed. This is the essential cause of the river-whiting phenomenon.

The sources of high concentrations of SO_4_^2-^ and H_2_S in the hypolimnetic water include not only direct SO_4_^2-^ and H_2_S input from the catchment, but also the sulfur-enriched surface sediments which may release H_2_S through the sulfate reduction processes. The contaminated sediment, as a result of persistent acid mine drainage input from the watershed, has acted as an important contamination source for sulfur to the overlying water, especially from the beginning of 2000s. This is why the river-whiting phenomenon occurred only in recent ten years. Following the development of water stratification in Aha Reservoir during June-October, the water below the thermocline is depleted of dissolved oxygen, and contains high levels of SO_4_^2-^ and hydrogen sulfide produced in strong reducing conditions. When sulfur-enriched hypolimnetic reservoir water drains into the river, SO_4_^2-^ concentration is increased as a result of the oxidation of H_2_S, and the solubility of CaSO_4_ decreases with increasing temperature, thus promoting the precipitation of CaSO_4_. This is why the river-whiting phenomenon occurred in the Xiaoche River normally from June to October.

It has been previously demonstrated that following the development of anoxia in the hypolimnion, the water quality conditions of discharge water may be dramatically altered [1, 19]. There are more than 50000 large dams in the world until now. Some of them discharge water from reservoirs with outflow intakes in the hypolimnion and some of them release the hypolimnetic water via seepage under the dam or through penstock just off the bottom. At reservoir’s earlier stage, discharge water may be clean. With the increase of reservoir age and the persistent accumulation of pollutants in reservoir system, discharge water may contain high levels of pollutants, especially in stratified reservoirs. This may lead to unpredicted disasters. Here we, for the first time, demonstrated a particular seasonal river-whiting phenomenon caused by seepage of hypolimnetic water from a seasonally stratified reservoir. More investigations are needed to illuminate the water quality condition of discharge water from reservoirs and estimate its impacts on the downstream eco-environment.
